# Supplementary material for: Bisphenol AF Promoted the Growth of Uterus and Activated Estrogen Signaling Related Targets in Various Tissues of Nude Mice with SK-BR-3 Xenograft Tumor
Source: Int J Environ Res Public Health. 2022 Nov 26;19(23):15743. doi: 10.3390/ijerph192315743 (PMC9741110; doi:10.3390/ijerph192315743)
Supplement: Supplementary file 1 [file ijerph-19-15743-s001.zip › ijerph-2028017-supplementary.pdf]

International Journal of
*Environmental Research
and Public Health*

MDPI

Supplementary Materials:

Table S1. Effect of BPAF on the weight (g, mean ± SD) of liver, kidney and hypothalamus from *Balb/cA* nude mice with SK-BR-3 xenograft tumor.

Group	Liver	Kidney	Hypothalamus
Normal control	1.122±0.194	0.299±0.045	0.0115±0.003
Bearing tumor control	1.173±0.146	0.313±0.020	0.014±0.006
20 mg/kg bw/day	1.259±0.084	0.3414±0.059	0.013±0.001
100 mg/kg bw/day	1.135±0.101	0.3054±0.022	0.013±0.005

Table S2. Effect of BPAF on organ coefficients (%, mean ± SD) of liver, kidney and hypothslamus from *Balb/cA* nude mice with SK-BR-3 xenograft tumor.

Group	Liver	Kidney	Hypothalamus
Normal control	5.564±0.627	1.488±0.132	0.058±0.015
Bearing tumor control	5.861±0.487	1.568±0.097	0.070±0.029
20 mg/kg bw/day	6.096±0.278	1.655±0.283	0.063±0.024
100 mg/kg bw/day	5.53±0.433	1.484±0.064	0.064±0.025

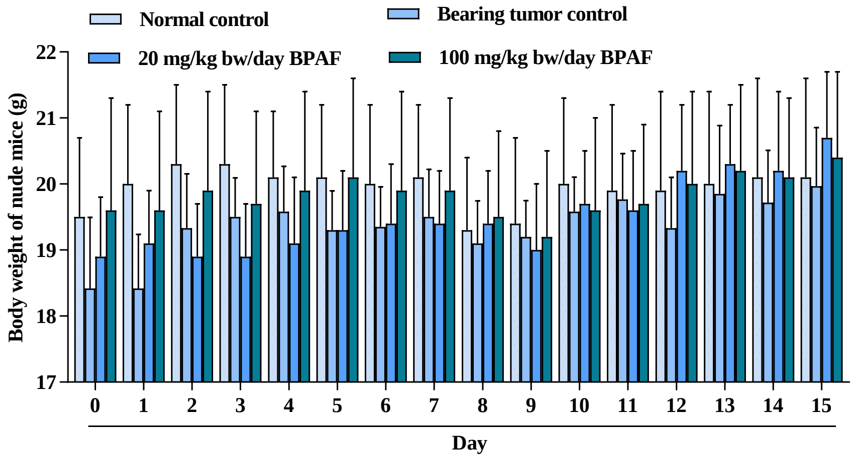

Figure S1. Effect of BPAF in different exposure days on body weight (g, mean ± SD) of *Balb/cA* nude mice with SK-BR-3 xenograft tumor.
